# Arrhythmic Risk in Carriers of Predicted Deleterious Rare Variants in Dilated and Arrhythmogenic Cardiomyopathy Genes

**DOI:** 10.1016/j.jacadv.2026.103066

**Published:** 2026-07-28

**Authors:** Ilaria Gandin, Andrea Mario Vergani, Michela Carlotta Massi, Alessia Paldino, Martina Setti, Maria Perotto, Marco Merlo, Gianfranco Sinagra, Giulia Barbati, Emanuele Di Angelantonio, Francesca Ieva, Matteo Dal Ferro

**Affiliations:** a Biostatistics Unit, Department of Medicine, Surgery and Health Sciences, University of Trieste, Trieste, Italy; b Department of Electronics, Information and Bioengineering (DEIB), Politecnico di Milano, Milan, Italy; c Department of Mathematics, Politecnico di Milano, Milan, Italy; d Health Data Science Centre, Human Technopole, Milan, Italy; e Cardiovascular Department, Azienda Sanitaria Universitaria Giuliano Isontina, Trieste, Italy; f Division of Cardiology, Department of Medicine, University of Verona, Verona, Italy; g Department of Medicine, Surgery and Health Sciences, University of Trieste, Trieste, Italy; h British Heart Foundation Cardiovascular Epidemiology Unit, Department of Public Health and Primary Care, University of Cambridge, Papworth Road, Cambridge Biomedical Campus, Cambridge, United Kingdom; i Victor Phillip Dahdaleh Heart and Lung Research Institute, University of Cambridge, Papworth Road, Cambridge Biomedical Campus, Cambridge, United Kingdom; j NIHR Blood and Transplant Research Unit in Donor Health and Behaviour, University of Cambridge, Cambridge, United Kingdom; k BHF Centre of Research Excellence, School of Clinical Medicine, University of Cambridge, Addenbrooke's Hospital, Cambridge, United Kingdom; l Health Data Research United Kingdom Cambridge, Wellcome Genome Campus and University of Cambridge, Cambridge, United Kingdom

**Keywords:** arrhythmogenic cardiomyopathy, dilated cardiomyopathy, rare variants, risk prediction, whole exome sequencing

## Abstract

**Background:**

In dilated (DCM) and arrhythmogenic cardiomyopathies (ACM), monogenic variants in causative genes are key prognostic factors. In the general population, the clinical role of these variants remains debated.

**Objectives:**

This study aimed to determine the association between rare, predicted deleterious variants (PDrV) in DCM- and ACM-associated genes and disease-related outcomes in the general population.

**Methods:**

Using United Kingdom Biobank whole-exome sequencing data, we identified PDrVs in 25 DCM/ACM-validated genes. We assessed disease penetrance in carriers and their risk for two primary outcomes—sudden cardiac death/malignant ventricular arrhythmias (SCD/MVA) and heart failure death/heart transplant (HF/HT)—using cause-specific Cox models accounting for competing risks.

**Results:**

Among 469,671 participants, 54.2% were females and the median age at baseline was 53.5 (IQR: 10.3). During a median follow-up of 14 years (IQR: 2), 5786 SCD/MVA and 4611 HF/HT events occurred. A PDrV was found in 12,973 (2.8%) individuals. Despite low penetrance for DCM (1.0%), PDrV impacted on both outcomes. Compared to noncarriers, PDrV carriers had a higher risk of SCD/MVA (HR: 1.28; 95% CI: 1.11-1.48). In participants free from DCM or other heart diseases at recruitment, SCD/MVA risk was solely associated with ACM genes (HR: 1.34; 95% CI: 1.07-1.69). Carriers also exhibited a higher risk of HF/HT (HR: 1.32; 95% CI: 1.08-1.62), which was not confirmed in subgroup without other heart diseases.

**Conclusions:**

PDrV carriers have a higher risk of severe cardiac events, even without a clinical overt disease phenotype at baseline evaluation. Moreover, PDrV in arrhythmic genes significantly influence SCD/MVA risk, regardless of phenotypic diagnosis of DCM.

## Highlights

• Predicted deleterious rare variants in dilated cardiomyopathy and arrhythmogenic cardiomyopathy genes increase the risk for severe cardiac events in the general population.
• Variants in arrhythmic genes contribute to the risk of life-threatening arrhythmic events in apparently healthy individuals.
• Investigating outcomes is the key since severe arrhythmic events can be the first disease manifestation.

Patients with cardiomyopathies are frequently found to be carriers of pathogenic or likely pathogenic (P/LP) variants in disease-associated genes. These monogenic variants are present both in familial and sporadic cases, showing autosomal dominant heritability. Penetrance is incomplete and phenotypic expression is highly variable. Notably, dilated cardiomyopathy (DCM) and arrhythmogenic cardiomyopathy (ACM) are characterized by significant overlaps in genetic background, clinical presentation, and outcomes. Although there is evidence supporting a complex genetic architecture,^1^ curated gene lists and gene-disease causative associations classified from moderate to definitive are available for these diseases.^2^, ^3^, ^4^

Data on variant penetrance are often limited, and when available, they typically come from clinical registries and family-based studies,^5^^,^^6^ showing that variant penetrance for cardiomyopathies is as high as 30% to 60% in first-degree relatives.

However, there is a growing interest in investigating the effect of putatively pathogenic variants in cardiomyopathy genes in the general population.^7^^,^^8^ Indeed, despite using different methodologies and outcomes, recent studies on the general population have collectively highlighted a low to very low disease penetrance of predicted deleterious rare variants (PDrVs) in cardiomyopathies associated genes.^7^, ^8^, ^9^, ^10^ In addition, data on whether a cardiomyopathy-related genotype is associated with distinct clinical outcomes in the general population—particularly major ventricular arrhythmias or sudden cardiac death (SCD)—remain scarce,^11^ often limited to analyses of composite outcomes^7^^,^^8^ or not considering the time-to-event information.^9^

The lack of such information is particularly relevant to the problem of “concealed cardiomyopathy”, in which sudden cardiac arrest or SCD occurs before any observable structural heart changes.^12^^,^^13^ In a recent study of autopsy-inconclusive SCDs, genetic testing revealed that, among P/LP variants in cardiomyopathy genes, nearly two-thirds were found in ACM-related genes.^14^ This finding supports the current understanding that the genotype plays a major role in disease prognosis.^15^ Variants in arrhythmia-associated genes, such as *LMNA*, *FLNC*, *PKP2*, *DSP*, and others,^1^^,^^16^^,^^17^ confer a significant risk of major ventricular arrhythmias regardless of the specific cardiomyopathy phenotype (DCM or arrhythmogenic right ventricular cardiomyopathy). Therefore, in these genotypes, arrhythmias may be a key determinant of both penetrance and outcome, even in the general population.

Understanding the impact of cardiomyopathy genotypes in the general population has become an urgent issue, especially in the context of “secondary findings”, that is, putative pathogenic variants identified through genetic testing that are not related to the primary clinical indication for testing. The widespread adoption of whole exome sequencing in several clinical contexts has drawn increased attention to the management of secondary findings. Currently, there is insufficient evidence to accurately assess the associated risks, and there are ongoing calls to address this problem and collect relevant information.^18^, ^19^, ^20^, ^21^

To investigate the role of rare, predicted deleterious variants in validated DCM-ACM genes in the general population, we analyzed carriers identified in the United Kingdom Biobank whole-exome sequencing data set comprising 470,000 participants. Our study aimed to assess the clinical impact of PDrV and evaluate the risk of specific disease-related outcomes, with a primary focus on ventricular arrhythmic events across different genotypes, regardless of their phenotype.

## Methods

### Study population

The United Kingdom Biobank study is a large-scale prospective cohort study, gathering comprehensive health data from over 500,000 United Kingdom participants aged between 40 and 69 at the time of recruitment.^22^ Thanks to the integration of comprehensive phenotyping conducted at assessment centers, linkage to primary and external hospital records, and analysis of biological samples (including genetic analysis), United Kingdom Biobank is one the most widely utilized resources in biomedical research. The study protocol is publicly accessible.^23^

The United Kingdom Biobank resource was approved by the United Kingdom Biobank Research Ethics Committee and all participants provided written informed consent to participate. This research has been conducted using the United Kingdom Biobank Resource under Application Number 82779.

### Variants selection

**Figure 1:**
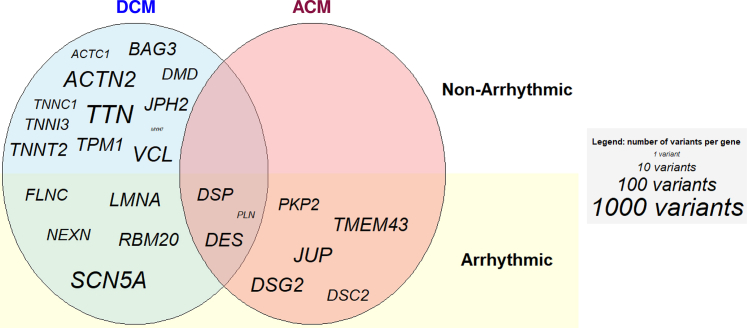
**Genes Considered in the Study** Genes are grouped by gene type (arrhythmic and nonarrhythmic) and associated disease (dilated cardiomyopathy [DCM] and arrhythmogenic cardiomyopathy [ACM]). The font size for gene names increases logarithmically with the number of subjects with mutation.

The identification of PDrV was based on whole exome sequencing data that were available for over 469,000 individuals within the cohort, for which the methodology has been previously described.^22^^,^^24^ For this study, 25 genes that have a moderate or higher level of evidence supporting their causal relationship with DCM and/or ACM based on most recent guidelines provided by expert panels were selected^2^, ^3^, ^4^ (Figure 1). As reported in Supplemental Table 1, genes were divided into arrhythmic or nonarrhythmic categories based on current evidence. To identify P/LP variants within these genes, a first filtering step retained only variants with allele frequency not exceeding established disease-specific maximum frequency thresholds^25^ (allele frequency ≤9.2e-5 in both gnomAD v3.1.2^26^ and United Kingdom Biobank). Second, for each gene, specific variant zigosity and classes (missense and/or predicted loss of function: nonsense, frameshift, and essential splice site variants) were included according to current knowledge of the gene's function and its role in disease (Supplemental Table 1). Missense variants were considered putatively deleterious only if reported deleterious by 5/5 prediction algorithms^22^ (SIFT,^27^ PolyPhen2 HDVI and PolyPhen2 HVAR,^28^ LRT,^29^ MutationTester).^30^ For the *TTN* gene, only exons with percentage spliced in >90% in cardiac-specific transcripts were included, as recommended.^31^ Individuals carrying at least 1 of the selected variants were considered carriers of putative P/LP variants (denoted by G+) otherwise they were considered gene elusive (denoted by G-).

### Phenotypes and outcome definitions

Phenotypes and outcomes were identified based on data from inpatient and outpatient diagnosis codes, procedure codes, death registry records, and medical history interviews. For the majority of participants (62.4%), health-related outcomes from electronic health record (EHR) were available for a period preceding the date of enrollment. The observation time spanned from the date of the earliest available EHR (or the date of enrollment if no prior records were available) to the most recent follow-up (dated October 2023).

To study the penetrance of PDrV, individuals with clinical DCM diagnosis were identified based on diagnostic codes or cause of death records (Supplemental Table 2). As diagnostic codes for ACM were not available in United Kingdom Biobank, penetrance analysis for this cardiomyopathy form could not be carried out.

In this study two primary composite outcomes were investigated: 1) SCD or malignant ventricular arrhythmias (MVAs); and 2) heart failure–related death or heart transplant (HF/HT). MVAs included ventricular fibrillation, sustained ventricular tachycardia, appropriate ICD interventions, and aborted SCD (Supplemental Tables 3 and 4). Secondary outcome of the study was all-cause mortality.

To conduct a subgroup analysis excluding individuals with other well-known conditions (outside of DCM and ACM) that could influence outcomes, additional phenotypes were identified and were grouped as “other heart diseases” (Supplemental Table 5). Specifically, these were ischemic heart disease (chronic and acute forms, including myocardial infarction, coronary artery bypass grafting, and coronary angioplasty with or without stenting) and hypertrophic cardiomyopathy (both obstructive and nonobstructive hypertrophic cardiomyopathy).

### Statistical analysis

Continuous variables were summarized with mean and SD (or median and IQR). Comparison of categorical variables was performed with chi-square or Fisher exact tests.

**Figure 2:**
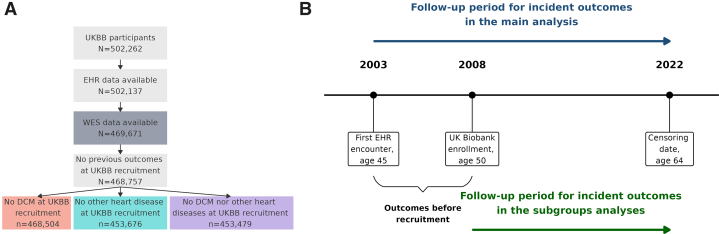
**Study Overview** (A) Study-flow diagram. After the exclusion of individuals without EHR or whole exome sequencing data, the remaining individuals were included in the main analyses comparing G+ vs G- groups. For the subgroup analyses, which were restricted to individuals free from DCM or other heart disease at United Kingdom Biobank enrollment, participants with a history of SCD/MVA or HF/HT before enrollment were also excluded. (B) Observational times for a sample participant that was enrolled in United Kingdom Biobank in 2008 but had first EHR record dated in 2003. For the main analysis comparing G+ and G-, the follow-up period for incident outcomes began on the date of the first EHR encounter. For subgroup analyses, if the subject was free from outcomes before recruitment, follow-up period started at United Kingdom Biobank enrollment. DCM = dilated cardiomyopathy; EHR = electronic health record; UKBB = United Kingdom Biobank; WES = whole-exome sequencing.

Time-to-event analysis was performed using age as timescale, as recommended in genetic studies.^32^ As United Kingdom Biobank provides phenotypic information from EHR data referring to a period before the enrollment date, each individual was observed from the earlier of either the first EHR encounter or the United Kingdom Biobank recruitment date (Figure 2); this allowed us to extend the time horizon of our analyses, whenever possible. However, because outcomes data were not available from birth, the analysis accounted for left truncated (by date of study entry) and right censoring. When estimating risk of SCD/MVA, other-cause mortality and HF/HT were considered competing events. Similarly, when estimating risk of HF/HT, other-cause mortality and SCD/MVA were considered competing events. For both outcomes of interest, a cause-specific Cox model was estimated using the counting-process setup, including sex, age, and genetic status (G+/G-) as independent variables. Only a limited set of covariates was considered, since limited information was available or subjects at the time of the first EHR encounter. The only variable with missing data was smoking status (0.5% missing), which was used in additional analysis for robustness; all models were fitted on complete cases. The proportional hazard assumption was assessed by analysis of Schoenfeld residuals (both formal test and visual inspection) and sensitivity analysis relaxing the assumption (see Supplemental Material). To provide more complete information, particularly regarding absolute risk prediction, Fine-Gray subdistribution models were also explored. The computation of cumulative incidence functions and relative 95% CIs accounted for both competing risks and left-truncation by obtaining the empirical transition matrix, along with the covariance estimator.^33^

As a sensitivity analysis of the role of PDrVs, additional models were estimated considering only individuals without clinically overt DCM or other heart diseases. In these subgroup analyses, follow-up began at the United Kingdom Biobank enrollment date to allow for the retrieval of information on prior diagnoses and clinical events. Individuals who had experienced the outcomes of interest before enrollment were excluded (Figure 2).

All statistical analyses were performed in R (version 4.4.3, R Foundation for Statistical Computing), using packages survival, tidyverse, ggsurvfit, etm.

## Results

### Study population

Population characteristics are reported in Table 1. Among 469,671 participants, 1,546 (0.3%) had DCM diagnosis and 36,538 (7.8%) had other heart diseases (ischemic heart disease n = 36,054, 7.7%; hypertrophic cardiomyopathy n = 674, 0.1%). In the cohort, 12,937 (2.8%) individuals were carriers of PDrV. Among them, 155 individuals carried PDrV in more than 1 gene and 41 individuals carried more than 1 PDrV in a single gene. PDrVs were found in all 25 genes except *MYH7* and *JUP*. Higher proportions of G+ were found for *SCN5A* (2,673, 20.7%), *TTN* (1965, 15.2%), *ACTN2* (1,392, 10.8%), *VCL* (1,381, 10.7%), and *DES* (663, 5.1%) genes. Mutation yield was almost equally distributed between arrhythmic genes (6,535, 50.5%) and nonarrhythmic genes (6,402, 49.5%).

**Table 1:** Clinical Characteristic of United Kingdom Biobank Cohort According to G+ and G- Status

	All (N = 469,671)	G+ (n = 12,937, 2.8%)	G- (n = 456,734, 97.2%)
Female, n (%)	254,553 (54.2%)	7,073 (54.7%)	247,480 (54.2%)
Age at first observation, median (IQR)	53.5 (10.3)	53.3 (10.3)	53.5 (10.3)
Age at recruitment, median (IQR)	58.0 (13.0)	58.0 (13.0)	58.0 (13.0)
Age at last follow-up, median (IQR)	72.0 (13.0)	71.0 (13.0)	72.0 (13.0)
Ethnicity, n (%)			
Asian	10,546 (2.2%)	557 (4.3%)	9,989 (2.2%)
Black	7,272 (1.5%)	297 (2.3%)	6,975 (1.5%)
Mixed	2,722 (0.6%)	121 (0.9%)	2,601 (0.6%)
White	442,763 (94.3%)	11,728 (90.7%)	431,035 (94.4%)
Other	4,172 (0.9%)	167 (1.3%)	4,005 (0.9%)
Unknown	2,196 (0.5%)	67 (0.5%)	2,129 (0.5%)
BMI [kg/m^2^],^a^ mean (SD)	27.4 (4.8)	27.4 (4.7)	27.4 (4.8)
BSA [m^2^],^a^^,^^b^ mean (SD)	1.9 (0.2)	1.9 (0.2)	1.9 (0.2)
Hypertension, n (%)^a^	125,601 (26.7%)	3,387 (26.2%)	122,214 (26.8%)
Diabetes, n (%)^a^	24,388 (5.2%)	704 (5.4%)	23,684 (5.2%)
COPD, n (%)^a^	9,095 (1.9%)	229 (1.8%)	8,866 (1.9%)
Creatinine [μmol/L],^a^ mean (SD)	72.3 (18.6)	72.0 (18.3)	72.3 (18.6)
Smoking status, n (%)^a^			
Current	49,376 (10.5%)	1,309 (10.1%)	48,067 (10.5%)
Previous	162,170 (34.5%)	4,398 (34.0%)	157,772 (34.5%)
Never	255,732 (54.4%)	7,159 (55.3%)	248,573 (54.4%)
Unknown	2,393 (0.5%)	71 (0.5%)	2,322 (0.5%)
DCM diagnosis or death, n (%)	1,546 (0.3%)	130 (1.0%)	1,416 (0.3%)
Other heart disease, n (%)	36,538 (7.8%)	1,069 (8.3%)	35,469 (7.8%)
Ischemic heart disease	36,054 (7.7%)	1,046 (8.1%)	35,008 (7.7%)
Hypertrophic cardiomyopathy	674 (0.1%)	26 (0.2%)	648 (0.1%)
Outcomes, n (%)			
All-cause death	41,204 (8.8%)	1,150 (8.9%)	40,054 (8.8%)
HF/HT	4,611 (1.0%)	164 (1.3%)	4,447 (1.0%)
SCD/MVA	5,786 (1.2%)	199 (1.5%)	5,587 (1.2%)

BMI = body mass index; BSA = body surface area; COPD = coronary obstructive pulmonary disease; DCM = dilated cardiomyopathy; HF/HT = heart failure–death; MVA = malignant ventricular arrhythmia; SCD = sudden cardiac death.

a At recruitment.

b BSA = 0.20247 x weight^0.425^ x height^0.725^ (weight in [kg], height in [m]).

Among 12,937 G+ individuals, 130 (1.0%) had DCM diagnosis. This proportion was significantly higher than in the G- group, in which DCM cases were 1,416 (0.3%, *P* < 0.001). Looking at single genes, penetrance for DCM was higher in *FLNC* (4.6%), *TTN* (3.6%), *ACTC1* (2.2%), *DMD* (2.0%), and *TNNI3* (1.5%). DCM penetrance was lower in arrhythmic genes (33/6,535 = 0.5%) compared to nonarrhythmic genes (97/6,402 = 1.5%; *P* < 0.001). Difference in penetrance was also found comparing genes with definitive evidence (105/8,248 = 1.3%) vs moderate evidence (30/4,699 = 0.6%; *P* < 0.001). Focusing on the genetic yield in DCM cases, a PDrV was found in 8.4% of DCM diagnoses. The more frequently associated genes with DCM were *TTN* (4.6%), *SCN5A* (0.6%), and *VCL* (0.5%).

### Impact of PDrV on clinical outcome in the United Kingdom Biobank population

**Figure 3:**
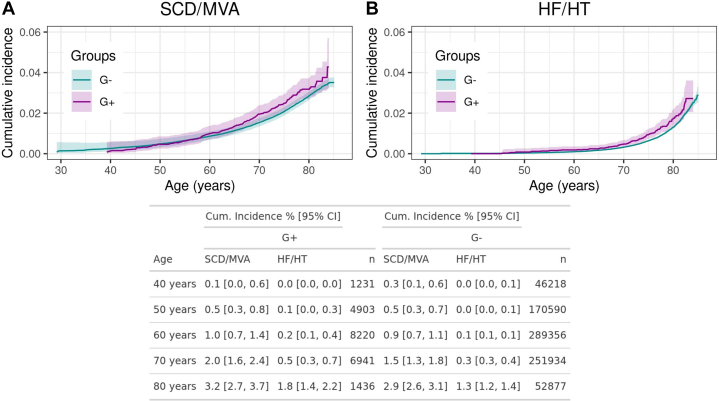
**Risk Curves by Genetic Status** Cumulative incidence curves for (A) SCD/MVA and (B) HF/HT stratified by genetic status (G+ vs G-). Shaded areas are 95% CIs. HF/HT = heart failure–related death/heart transplant; MVA = malignant ventricular arrhythmias; SCD = sudden cardiac death.

During a median follow-up of 14 years (IQR: 2), 5,786 individuals experienced SCD/MVA, 4,611 experienced HF/HT ,and 41,204 died. By age 80, cumulative incidence of SCD/MVA was 3.2% (95% CI: 2.7-3.7) in G+ group and 2.9% (95% CI: 2.6-3.1) in the G- group, an absolute difference of 0.3 percentage points. At the same age, the risk of HF/HT was 1.8% (95% CI: 1.4-2.2) in G+ group and 1.3% (95% CI: 1.2-1.4) in the G- group, an absolute difference of 0.5 percentage points (Figure 3).

**Figure 4:**
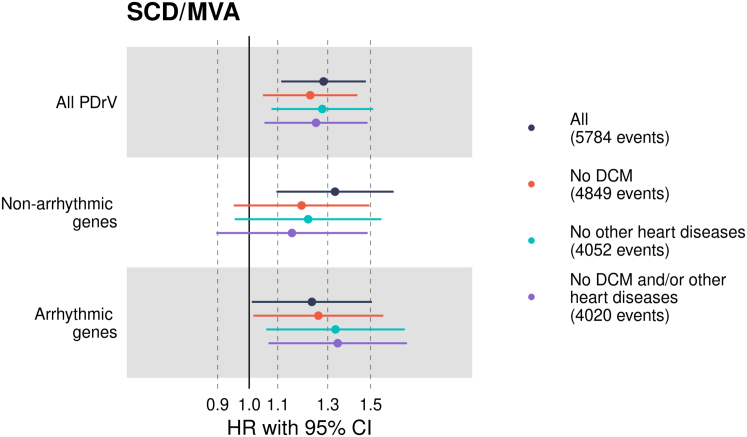
**Results of the Multivariable Model for SCD/MVA Outcome** The three blocks (“all PDrV”, “nonarrhythmic genes”, and “arrhythmic genes”) indicate the comparison groups, with G- serving as the reference in all analyses. Colors specify whether the results refer to the main analysis or to the subgroup analyses, in which individuals with DCM, other heart diseases, or both were progressively excluded. PdrV = predicted deleterious rare variants; other abbreviations as in Figures 2 and 3.

The age-adjusted multivariable model proved that G+ individuals were at higher risk for SCD/MVA compared to noncarriers (HR: 1.28; 95% CI: 1.11, 1.48; *P* < 0.001). Consistent results were observed in subgroup analyses, in which participants with DCM, other heart diseases, or both were progressively excluded (Figure 4, Supplemental Table 6).

**Figure 5:**
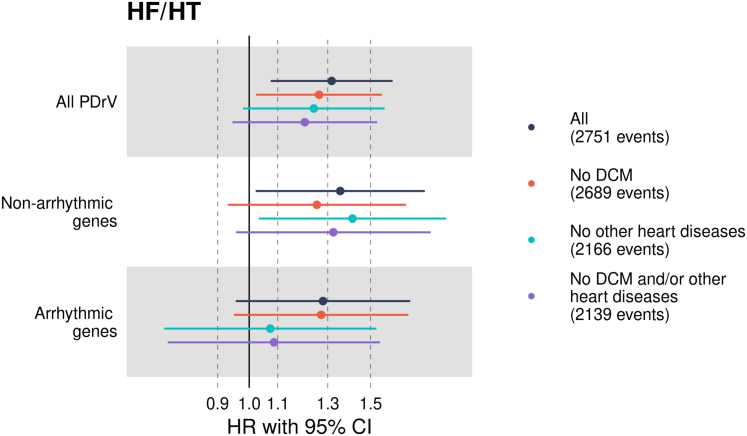
**Results of the Multivariable Model for HF/HT Outcome** The three blocks (“all PDrV”, “nonarrhythmic genes”, and “arrhythmic genes”) indicate the comparison groups, with G- serving as the reference in all analyses. Colors specify whether the results refer to the main analysis or to the subgroup analyses, in which individuals with DCM, other heart diseases, or both were progressively excluded. Abbreviations as in Figures 2, 3, and 4.

As a second step, the impact of arrhythmic and nonarrhythmic genes was investigated. Compared to the G- group, the increased risk of SCD/MVA in the G+ group was similar for nonarrhythmic gene variants (HR: 1.33; 95% CI: 1.10-1.62) (see Figure 5) and arrhythmic gene variants (HR: 1.23; 95% CI: 1.01-1.50).

This association was further investigated with subgroup analyses (Figure 4, Supplemental Table 6). After excluding participants with DCM or prior outcomes at the time of United Kingdom Biobank recruitment (1,167 individuals and 935 SCD/MVA events excluded), the association of SCD/MVA with nonarrhythmic genes was no longer statistically significant (HR: 1.19; 95% CI: 0.95-1.49), whereas the effect estimate for arrhythmic genes remained significant (HR: 1.26; 95% CI: 1.01-1.57). Moreover, when individuals with other heart disease were also excluded (15,995 individuals and 1732 events excluded), in our sample, the point estimate of the risk associated with variants in arrhythmic genes increased (HR: 1.34; 95% CI: 1.07-1.69), although the difference between this and the previous estimates was not statistically significant.

Carrying a PDrV also showed an association with the HF/HT outcome: HF/HT risk was higher in the G+ compared to the G- group (HR: 1.32; 95% CI: 1.07-1.61; *P* = 0.008). In this case, the association was no longer significant after excluding participants with other heart diseases (see Figure 5 and Supplemental Table 8).

When analyzing the impact of arrhythmic and nonarrhythmic genes separately, a significant association was found only for PDrV in nonarrhythmic genes (HR: 1.36; 95% CI: 1.02-1.80) (Supplemental Table 8). After excluding individuals with DCM, the association did not reach statistical significance.

For both SCD/MVA and HF/HT outcomes, in the subgroups of individuals without DCM and/or other heart disease, analyses including hypertension and smoking status yielded similar estimates of the PDrV effect (Supplemental Tables 7 and 9).

Carrying a PDrV was not associated with an increased risk of all-cause mortality (HR: 1.04; 95% CI: 0.98- 1.10; *P* = 0.23). Since this result differs from previous studies that reported a significant impact of PDrV in DCM genes on all-cause mortality (HR: 1.14; 95% CI: 1.01-1.27; in Asatryan et al^8^), additional analyses were performed selecting only variants from the same gene list and no association was observed (Supplemental Table 10).

Fine-Gray subdistribution HRs, providing a complementary perspective on cumulative incidence, were also estimated for both outcomes and showed good consistency with the cause-specific estimates (Supplemental Tables 11 and 12).

## Discussion

This study used the 470,000 exome-sequencing United Kingdom Biobank data to evaluate the impact of PDrV in genes associated with DCM and/or ACM on 2 selected clinical outcomes, within the general population. Our key findings are the following: 1) individuals carrying PDrV showed a relative risk of severe arrhythmic events increased by 23% to 34% compared to noncarriers; and 2) being carrier of PDrV in arrhythmic genes was associated with a higher risk of SCD/MVA regardless of DCM diagnosis (Central Illustration).

**Central Illustration:**
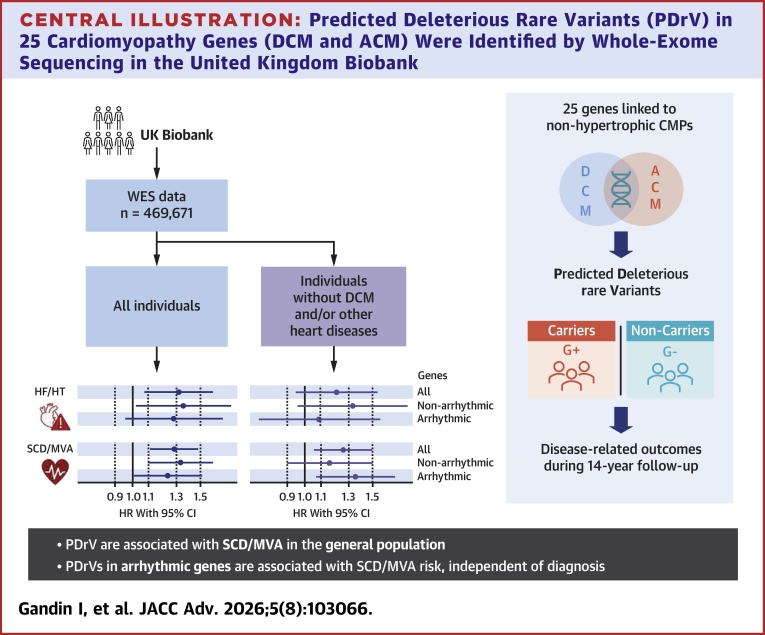
**Predicted Deleterious Rare Variants (PDrV) in 25 Cardiomyopathy Genes (DCM and** ACM**) Were Identified by Whole-Exome Sequencing in theUnited KingdomBiobank** Carriers (G+) and noncarriers (G-) were compared for HF/HT and SCD/MVA over a median 14-year follow-up. PDrV were associated with SCD/MVA, and variants in arrhythmic genes with SCD/MVA independently of diagnosis. ACM = arrhythmogenic cardiomyopathy; CMP = cardiomyopathy; DCM = dilated cardiomyopathy; HF/HT = heart failure–related death/heart transplant; MVA = malignant ventricular arrhythmias; SCD = sudden cardiac death; WES = whole-exome sequencing.

### Disease expression of PDrV

Most information on the impact of rare variants in Mendelian diseases has been derived from referral or registry populations, but the applicability of these high-penetrance estimates to the general population has been widely challenged. The recent release of about 500,000 exomes by the United Kingdom Biobank provides a unique opportunity to refine the assessment of the clinical role of these rare variants in the largest available population data set available to date.

The DCM prevalence of 1% in G+ group observed in this work should be considered very low; however, it parallels the 0.24% to 3.1% from previous works (0.24% to 1.42% in Shah et al;^7^ 1.79% in Asatryan et al,^8^ 1.2% to 3.1% in Bourfiss et al^9^), which used a previous release of United Kingdom Biobank data including whole exome sequencing results for half of the cohort. Although EHR-linked biobanks provide invaluable resources, featuring large longitudinal cohorts and vast amounts of heterogeneous data, the United Kingdom Biobank likely underrepresents the prevalence of cardiomyopathy diagnoses due to both “healthy volunteer” selection bias^34^ and under-reporting of disease.^35^ In this setting, variants with high penetrance, early onset, sex-related effects, familial oligo/polygenic backgrounds, and environmental triggers leading to earlier and more severe cardiomyopathy manifestations are likely to be under-represented. Our approach in selecting PDrV aligns with the methodology previously described by Asatryan et al. We believe this approach better reflects a real-world clinical scenario, where a PDrV could be identified as a secondary finding, rather than focusing exclusively on high-evidence, already described variants.^9^ Unfortunately, the current study could only investigate penetrance for DCM and not for ACM, as the latter is not identified by ICD-10 diagnostic codes, and the additional cardiac phenotypic information available for United Kingdom Biobank participants (cardiac magnetic resonance imaging and electrocardiogram) is not sufficient to accurately identify ACM cases.

Given the limitations in studying cardiomyopathy diagnoses and phenotypes in an EHR-linked biobank, our study was primarily focused on the impact of PDrV on clinical outcomes. For the SCD/MVA endpoint, we observed an increased risk associated with PDrV in both arrhythmic and nonarrhythmic genes. As shown in recent studies of SCD cases,^14^^,^^36^ individuals with unexplained SCD and no structural heart changes were enriched for P/LP variants also in nonarrhythmic genes, whose role remains uncertain. The observed increase in SCD/MVA risk among individuals without a diagnosis of DCM or other heart diseases (particularly ischemic heart disease) is clinically valuable. It is well known that SCD/MVA may represent the only manifestation of disease, especially for certain genotypes. This finding has direct clinical implication, suggesting that individuals identified as PDrV carriers may benefit from tailored cardiological follow-up strategies, including the use of Holter electrocardiogram monitoring. In the absence of overt disease, periodic follow-up may be considered. This information could also be useful in optimizing resource allocation for individuals with secondary findings, although further cost-effectiveness analyses are warranted.

A clear picture on the impact of PDrV on HF/HT outcome was not obtained in this study, as the association was not confirmed when the analysis was restricted to individuals without other heart disease. It is important to note that the HF endpoint represented a very advanced stage of the condition (leading to death or transplantation), which is more likely to occur after DCM or other heart disease diagnosis. Therefore, their exclusion may point to a potential mediation effect.

### Role of arrhythmic genes

Although the distinct roles of arrhythmic and nonarrhythmic genes in clinical contexts is increasingly recognized, the contribution of the two gene groups to cardiac events in the general population remain only partially elucidated in our analyses. As expected, for HF events, an increased risk was mostly related to PDrV in nonarrhythmic genes. Instead, PDrV in both arrhythmic and nonarrhythmic genes resulted associated with SCD/MVA risk.

In the subgroup analyses that included only apparently healthy individuals, the risk of SCD/MVA was found to be associated with PDrV in arrhythmic genes, whereas no association was observed for PDrV in nonarrhythmic genes. This change suggests that the arrhythmic risk of PDrV in these genes (mostly *TTN*) is linked to phenotypic manifestation (DCM). Similarly, PDrV in nonarrhythmic genes showed no association with HF/HT after excluding individuals with overt DCM, likely reflecting the higher penetrance observed in this gene group.

The trend observed in these subgroup analyses may suggest that PDrV in arrhythmic genes are particularly relevant in nonhypertrophic cardiomyopathies where SCD/MVA can be the first clinical manifestation, but it ought to be underlined that subgroup analyses resulted in a reduced sample size and thus in lower risk estimate precision.

### Strengths

This study has several strengths. First, it is the first to investigate the role of cardiomyopathy rare variants in the entire United Kingdom Biobank cohort, which includes approximately 470,000 individuals. Second, clinical outcomes were analyzed by distinguishing between arrhythmic events and HF-related events, providing a more detailed assessment of the impact of variants compared to previous studies. Third, in line with recent findings highlighting the importance of genotype-based classification of cardiomyopathies, our gene list included those associated with both DCM and ACM, improving the power of the analysis. Fourth, thanks to the large sample size, we were able to investigate the contribution of both arrhythmic and nonarrhythmic genes.

### Study Limitations

A number of limitations need to be highlighted. The primary limitation is the definition of PDrV. We applied very strict criteria for their selection, which likely led to the exclusion of some P/LP variants, such as rare missense variants in *MYH7* predicted deleterious by <5 algorithms. This may have caused some true carriers to be classified as noncarriers (nondifferential exposure misclassification), which would tend to weaken the observed associations.

When evaluating the penetrance of the selected variants, ACM forms were not considered due to the absence of corresponding diagnostic code in the United Kingdom Biobank. As for DCM forms, phenotypes were identified solely through clinical diagnoses or causes of death. However, previous studies have shown that mutations also impact subclinical forms of DCM that may be clinically silent, hence not recognized.^7^^,^^8^

Another limitation concerns the interpretation of the subgroup analysis results. Restricting these analyses to participants who are free, at study entry, from diseases that the genetic exposure may itself influence, can induce selection bias. Therefore, the observed attenuation of associations can only be hypothesized to result from mediation, since selection effects cannot be ruled out.

## Conclusions

Collectively, our findings confirm that being a carrier of PDrV does impact the risk of severe cardiac events in the general population. PDrVs in arrhythmic, high confidence genes significantly affect the risk of SCD/MVA regardless of the presence of phenotypic diagnosis of DCM. Such results contribute to the growing body of evidence supporting the clinical management of secondary genetic findings. Further studies are needed to investigate the reasons behind differences in penetrance of these variants among general population and patients’ cohorts.

Perspectives **COMPETENCY MEDICAL KNOWLEDGE:** This study shows that rare, predicted deleterious variants in cardiomyopathy genes influence the risk of severe cardiac events in the general population. Variants in arrhythmic genes are significantly associated with an increased risk of life-threatening arrhythmic events, regardless of a phenotypic diagnosis.

**TRANSLATIONAL OUTLOOK:** The increased arrhythmic risk found in apparently healthy carriers may suggest that variants in arrhythmic genes are particularly relevant in DCM and ACM where severe arrhythmic events can be the first clinical manifestation.

## Funding support and author disclosures

This research has been conducted using the United Kingdom Biobank Resource under Application Number 82779. Drs Vergani and Ieva acknowledge the support by MUR, grant Department of Excellence 2023 to 2027 (Department of Mathematics, Politecnico di Milano, Milano, Italy). All authors have reported that they have no relationships relevant to the contents of this paper to disclose.
