# Supplementary material for: Arrhythmic Risk in Carriers of Predicted Deleterious Rare Variants in Dilated and Arrhythmogenic Cardiomyopathy Genes
Source: JACC Adv. 2026 Jul 28;5(8):103066. doi: 10.1016/j.jacadv.2026.103066 (PMC13430186; doi:10.1016/j.jacadv.2026.103066)
Supplement: Supplementary material [file mmc1.pdf]

Arrhythmic risk in carriers of predicted deleterious rare variants in dilated and arrhythmogenic cardiomyopathy genes

Supplemental Material

Time to event modeling and competing risks

The proportional hazards assumption was investigated through several approaches. First, Schoenfeld residuals were visually inspected. Second, a formal statistical test was applied. For the variable of interest (the binary indicator for G+/G-), there was no evidence of departures from proportionality. Instead, the test indicated departures from proportionality for some of the covariates (sex and age). However, at our sample size this test was highly sensitive and could detect departures that were too small to be of practical relevance. To confirm that this did not affect our inference, we refitted the model allowing the effects of these covariates to vary across age strata ([40,55) years, [55,70) years, [70,85] years). The hazard ratio for G+ was essentially unchanged compared with the primary model (differences < 0.01). We therefore interpreted these results as non-proportionality of the covariates that does not influence the estimate of the genetic effect.

Given the etiologic focus of our study, cause-specific Cox models for competing risks provided the main results. However, to obtain additional information on absolute risk, the hazard ratios from Fine-Gray subdistribution models are useful, since the cause-specific hazard alone does not determine the cumulative incidence in the presence of competing risks, whereas the subdistribution hazard has a direct correspondence with the cumulative incidence function and thus informs absolute risk.

The subdistribution hazard ratios are reported in Tables S11 and S12. These were consistent in direction with the estimates from the cause-specific Cox models.

Genotypes and phenotypes definitions

Gene	Mutation	Evidence for DCM	Evidence for ARVC	Gene category	G+ individuals	DCM diagnosis
ACTC1	Missense or pLoF	Moderate/ Definite		Non-arrhythmic	46	1
ACTN2	Missense or pLoF	Moderate/ Definite		Non-arrhythmic	1392	6
BAG3	Missense or pLoF	Moderate/ Definite		Non-arrhythmic	307	2
DES	Missense or pLoF	Moderate/ Definite	Moderate/ Definite	Arrhythmic	663	4
DMD	pLoF	Moderate/ Definite		Non-arrhythmic	148	3
DSC2	Missense or pLoF		Moderate/ Definite	Arrhythmic	190	0
DSG2	Missense or pLoF		Moderate/ Definite	Arrhythmic	512	0
DSP	pLoF	Moderate/ Definite	Moderate/ Definite	Arrhythmic	232	3
FLNC	pLoF	Moderate/ Definite		Arrhythmic	87	4
JPH2	Missense or pLoF	Moderate/ Definite		Non-arrhythmic	475	1
JUP	Homozygous missense or homozygous pLoF		Moderate/ Definite	Arrhythmic	0	0
LMNA	Missense or pLoF*	Moderate/ Definite		Arrhythmic	696	5
MYH7	Missense	Moderate/ Definite		Non-arrhythmic	0	0

NEXN	pLoF	Moderate/ Definite		Arrhythmic	283	0
PKP2	pLoF		Moderate/ Definite	Arrhythmic	155	0
PLN	pLoF	Moderate/ Definite	Moderate/ Definite	Arrhythmic	8	0
RBM20	Missense or pLoF	Moderate/ Definite		Arrhythmic	514	6
SCN5A	Missense or pLoF*	Moderate/ Definite		Arrhythmic	2673	10
TMEM43	Missense or pLoF		Moderate/ Definite	Arrhythmic	559	2
TNNC1	Missense or pLoF	Moderate/ Definite		Non-arrhythmic	73	1
TNNI3	Missense or pLoF	Moderate/ Definite		Non-arrhythmic	137	2
TNNT2	Missense or pLoF	Moderate/ Definite		Non-arrhythmic	277	1
TPM1	Missense or pLoF	Moderate/ Definite		Non-arrhythmic	322	4
TTN	pLoF	Moderate/ Definite		Non-arrhythmic	1965	71
VCL	Missense or pLoF	Moderate/ Definite		Non-arrhythmic	1381	8

Table S1. List of genes (*only pLOF considered with high evidence).

Phenotype	Field names	Data codes
DCM	Diagnoses - main ICD10	I42.0
DCM	Diagnoses – secondary ICD10	I42.0
DCM	Underlying (primary) cause of death:ICD10	I42.0
DCM	Contributory (secondary) cause of death: ICD10	I42.0

Table S2. List of phenotypes used for the definition of DCM diagnosis.

Phenotype	Field names	Data codes
Reentry ventricular arrhythmia	Diagnoses (main and secondary)	I47.0
Ventricular tachycardia	Diagnoses (main and secondary), cause of death(primary and secondary)	I47.2
Cardiac arrest with successful resuscitation	Diagnoses (main and secondary)	I46.0
Cardiac arrest, unspecified	Diagnoses (main and secondary), cause of death (primary and secondary)	I46.9
Sudden cardiac death, so described	Diagnoses (main and secondary), cause of death(primary and secondary)	I46.1
Ventricular fibrillation and flutter	Diagnoses (main and secondary)	I49.0
Percutaneous transluminal ablation of ventricular wall	Operative procedures (main and secondary)	K57.6
Percutaneous radiofrequency ablation of epicardium	Operative procedures (main and secondary)	K64.1
Epicardial excision of rhythmogenic focus	Operative procedures (main and secondary)	K52.2
Endocardial excision of rhythmogenic focus	Operative procedures (main and secondary)	K52.3
Advanced cardiac pulmonary resuscitation	Operative procedures (main and secondary)	X50.3
External ventricular defibrillation	Operative procedures (main and secondary)	X50.4
Other specified external resuscitation	Operative procedures (main and secondary)	X50.8
Unspecified external resuscitation	Operative procedures (main and secondary)	X50.9

Table S3. List of phenotypes used for the definition of SCD/MVA outcome.

Phenotype	Field names	Data codes
Allotransplantation of heart and lung	Operative procedures (main and secondary)	K01.1
Allotransplantation of heart NEC	Operative procedures (main and secondary)	K02.1
Congestive heart failure	cause of death (primary and secondary)	I50.0
Left ventricular failure	cause of death (primary and secondary)	I50.1
Heart failure, unspecified	cause of death (primary and secondary)	I50.9

hypertensive heart disease	cause of death (primary and secondary)	I11.0, I11.9
Dilated cardiomyopathy	cause of death (primary and secondary)	I42.0
Alcoholic cardiomyopathy	cause of death (primary and secondary)	I42.6
Other cardiomyopathies	cause of death (primary and secondary)	I42.8
Cardiomyopathy, unspecified<br>Cardiac arrest, unspecified	cause of death (primary and secondary)<br>Diagnoses (main and secondary), cause of death (primary and secondary)	I42.9<br>I46.9
Open implantation of ventricular assist device	Operative procedures (main and secondary)	K54.1
Other specified open heart assist operations	Operative procedures (main and secondary)	K54.8

Table S4. List of phenotypes used for the definition of HF/HT outcome.

Phenotype	Field names	Data codes
Myocardial infarction		I21.X, I22.X, I23.X, I24.1, I24.2
Heart attack/myocardial infarction, Coronary artery bypass grafting, coronary angioplasty with or without stenting		1075, K40.1–40.4, K41.1–41.4, K45.1–45.5, K49.1–49.2, K49.8–49.9, K50.2, K75.1–75.4, K75.8–75.9
coronary angioplasty (ptca) +/- stent, coronary artery bypass grafts (cabg)		1070, 1095
Obstructive hypertrophic cardiomyopathy, other hypertrophic cardiomyopathy		I42.1, I42.2

Table S5. List of phenotypes used for the definition of other heart disease.

Subjects in analysis	Group of comparison (G- reference)	HR	95% CI lower	95% CI Upper
all	G+	**1.28**	**1.11**	**1.48**
excluding DCM	G+	**1.23**	**1.05**	**1.44**
excluding other heart disease	G+	**1.28**	**1.08**	**1.51**
excluding DCM and other heart disease	G+	**1.25**	**1.05**	**1.49**
only males	G+	**1.23**	**1.04**	**1.47**

only females	G+	**1.39**	**1.09**	**1.78**
all	non-arrhythmic	**1.33**	**1.10**	**1.62**
excluding DCM	non-arrhythmic	1.19	0.95	1.49
excluding other heart disease	non-arrhythmic	1.22	0.95	1.56
excluding DCM and other heart disease	non-arrhythmic	1.15	0.90	1.49
all	arrhythmic	**1.23**	**1.01**	**1.51**
excluding DCM	arrhythmic	**1.26**	**1.01**	**1.57**
excluding other heart disease	arrhythmic	**1.33**	**1.06**	**1.68**
excluding DCM and other heart disease	arrhythmic	**1.34**	**1.07**	**1.69**

Table S6. Cox proportional hazards regression comparing the risk of SCD/MVA between carrier groups ("Group of comparison") and G- subjects across subgroups ("Subjects in analysis").

Variable	HR	95% CI lower	95% CI Upper
Male	**2.29**	**2.14**	**2.44**
Age	**0.97**	**0.96**	**0.98**
Smoke	**1.31**	**1.23**	**1.40**
Hypertension	**1.62**	**1.53**	**1.73**
PDrV carrier	**1.25**	**1.06**	**1.49**

Table S7. Results of multivariable regression models for SCD/MVA in the subgroup of individuals without DCM and/or other heart disease. In this analysis, 2393 individuals were excluded beacuse not all covariates were measured.

Subjects in analysis	Group of comparison (G- reference)	HR	95% CI lower	95% CI Upper
all	G+	**1.32**	**1.07**	**1.61**
excluding DCM	G+	**1.26**	**1.02**	**1.56**
excluding other heart disease	G+	1.24	0.98	1.57

excluding DCM and other heart disease	G+	1.20	0.95	1.53
only males	G+	**1.32**	**1.03**	**1.68**
only females	G+	1.31	0.91	1.90
all	non-arrhythmic	**1.36**	**1.02**	**1.80**
excluding DCM	non-arrhythmic	1.25	0.93	1.69
excluding other heart disease	non-arrhythmic	**1.41**	**1.03**	**1.93**
excluding DCM and other heart disease	non-arrhythmic	1.32	0.96	1.83
all	arrhythmic	1.28	0.96	1.71
excluding DCM	arrhythmic	1.27	0.95	1.70
excluding other heart disease	arrhythmic	1.07	0.75	1.53
excluding DCM and other heart disease	arrhythmic	1.09	0.76	1.55

Table S8. Cox proportional hazards regression comparing the risk of HF/HT between carrier groups ("Group of comparison") and G– subjects across subgroups ("Subjects in analysis").

Variable	HR	95% CI lower	95% CI Upper
Male	**2.19**	**2.00**	**2.40**
Age	**0.94**	**0.93**	**0.95**
Hypertension	**2.60**	**2.38**	**2.83**
Smoke	**1.69**	**1.55**	**1.85**
PDrV carrier	1.22	0.96	1.55

Table S9. Results of multivariable regression models for HF/HT in the subgroup of individuals without DCM and/or other heart disease. In this analysis, 2393 individuals were excluded beacuse not all covariates were measured.

Subjects in analysis	Group of comparison (G- reference)	HR	95% CI lower	95% CI Upper
all	G+	1.04	0.98	1.10

excluding DCM	G+	1.03	0.97	1.09
excluding other heart disease	G+	1.02	0.96	1.09
excluding DCM and other heart disease	G+	1.02	0.96	1.09
all	Carriers of PDrV in the gene list of Asatryan et al.	1.04	0.98	1.11

Table S10. All-cause death results.

		Cause-spefic HR			Subdistribution HR		
**Subjects in analysis**	**Group of comparison (G- reference)**	**HR**	**95% CI lower**	**95% CI Upper**	**HR**	**95% CI lower**	**95% CI Upper**
all	G+	**1.28**	**1.11**	**1.48**	**1.28**	**1.11**	**1.47**
excluding DCM	G+	**1.23**	**1.05**	**1.44**	**1.22**	**1.04**	**1.43**
excluding other heart disease	G+	**1.28**	**1.08**	**1.51**	**1.28**	**1.08**	**1.51**
excluding DCM and other heart disease	G+	**1.25**	**1.05**	**1.49**	**1.25**	**1.05**	**1.48**
all	non-arrhythmic	**1.33**	**1.10**	**1.62**	**1.33**	**1.09**	**1.62**
excluding DCM	non-arrhythmic	1.19	0.95	1.49	1.19	0.95	1.49
excluding other heart disease	non-arrhythmic	1.22	0.95	1.56	1.21	0.95	1.55
excluding DCM and other heart disease	non-arrhythmic	1.15	0.90	1.49	1.15	0.89	1.48
all	arrhythmic	**1.23**	**1.01**	**1.51**	**1.23**	**1.01**	**1.50**
excluding DCM	arrhythmic	**1.26**	**1.01**	**1.57**	**1.26**	**1.01**	**1.56**
excluding other heart disease	arrhythmic	**1.33**	**1.06**	**1.68**	**1.34**	**1.06**	**1.68**
excluding DCM and other heart	arrhythmic	**1.34**	**1.07**	**1.69**	**1.35**	**1.07**	**1.70**

disease							

Table S11. Cause-specific and subdistribution (Fine-Gray) hazard ratios for the association between being carrier (“Group of comparison”) and SCD/MVA across subgroups (“Subjects in analysis”).

		Cause-spefic HR			Subdistribution HR		
**Subjects in analysis**	**Group of comparison (G- reference)**	**HR**	**95% CI lower**	**95% CI Upper**	**HR**	**95% CI lower**	**95% CI Upper**
all	G+	**1.32**	**1.07**	**1.61**	**1.31**	**1.07**	**1.61**
excluding DCM	G+	**1.26**	**1.02**	**1.56**	**1.26**	**1.02**	**1.55**
excluding other heart disease	G+	1.24	0.98	1.57	1.24	0.98	1.56
excluding DCM and other heart disease	G+	1.20	0.95	1.53	1.20	0.94	1.53
all	non-arrhythmic	**1.36**	**1.02**	**1.80**	**1.36**	**1.02**	**1.80**
excluding DCM	non-arrhythmic	1.25	0.93	1.69	1.25	0.93	1.68
excluding other heart disease	non-arrhythmic	**1.41**	**1.03**	**1.93**	**1.41**	**1.03**	**1.92**
excluding DCM and other heart disease	non-arrhythmic	1.32	0.96	1.83	1.32	0.95	1.83
all	arrhythmic	1.28	0.96	1.71	1.27	0.95	1.69
excluding DCM	arrhythmic	1.27	0.95	1.70	1.27	0.95	1.69
excluding other heart disease	arrhythmic	1.07	0.75	1.53	1.07	0.75	1.52
excluding DCM and other heart disease	arrhythmic	1.09	0.76	1.55	1.08	0.76	1.54

Table S12. Cause-specific and subdistribution (Fine-Gray) hazard ratios for the association between being carrier (“Group of comparison”) and HF/HT across subgroups (“Subjects in analysis”).
